# Touching Technology—Parents’ Experiences of Remote Consultations for Children With Severe Congenital Cardiac Conditions: Quasi-Experimental Cohort Study

**DOI:** 10.2196/54598

**Published:** 2024-10-22

**Authors:** Julie Elizabeth May McCullough, Marlene Sinclair, Jonathan Gillender, Brian McCrossan, Paul F Slater, Rosie Browne, Frank Casey

**Affiliations:** 1 Institute of Nursing and Health Research, Ulster University, York Street, Belfast, BT15 1ED, United Kingdom; 2 Department of Paediatric Cardiology, Royal Belfast Hospital for Sick Children, 180 Falls Road, Belfast, BT12 6BE, United Kingdom, 44 2890635350

**Keywords:** congenital heart disease, pediatric cardiology, pediatric cardiologist, pediatric, parent, digital health, digital technology, digital intervention, telemedicine, telehealth, virtual care, virtual health, virtual medicine, remote consultation, telephone consultation, video consultation, remote patient monitoring, technology acceptance, videoconferencing consultations

## Abstract

**Background:**

Remote consultations (RCs) using videoconferencing was recommended by the General Medical Council as the method for clinicians to provide patient consultations during the COVID-19 pandemic. Facilitating this while providing high-quality care depends on the usability and acceptability of the technology.

**Objective:**

This project aimed to investigate parents’ experiences of using videoconferencing technology for real-time RCs with children who had congenital heart defects during the COVID-19 pandemic lockdown.

**Methods:**

This study’s design was quasi-experimental and was underpinned by the Unified Theory of Acceptance and Use of Technology model that seeks to explain and predict an individual’s intention to use a technology. Parents were informed of this study by the medical team, posters were made available in the wards and clinics, and leaflets were left for browsing. Clinician screening of potential participants led to the identification of 33 children and parents who were enrolled on this study. The intervention was a web-based RC by medical staff using a secure, interactive videoconferencing platform (Pexip). Each child and their mother or father received 8 RCs with the same specialist doctor or nurse. Measurements were taken using web-based questionnaires pre and post consultation at the first, middle, and last events; questions were focused on the acceptability, usability, and clinical applicability of RCs. Parents’ experiences were explored using recorded interviews and analyzed thematically.

**Results:**

In total, 29 children aged 4‐1052 (mean 95, SD 191.14) days completed the project, receiving a total of 189 RCs as part of their routine care. Parents’ prior experience of consultation via videoconference was low; however, as time progressed, their use and acceptance of the technology increased. The intervention was warmly received by all parents who found the face-to-face component particularly useful for discussion with their child’s medical team. Furthermore, parents noted the savings on time, money, and childcare.

**Conclusions:**

While in-person consultations are considered the gold standard of patient care, increasing pressures on health services and staff reduce availability. Given the ease of access and additional benefits experienced by parents and their children, it is proposed that hybrid models of consultation and care provision are equal, if not superior, to in-person consultations in the management of children with severe congenital heart defects while reducing costs and pressure on the health service and parents.

## Introduction

The Royal Belfast Hospital for Sick Children (RBHSC) is the only dedicated center for pediatric congenital heart disease (CHD) in Northern Ireland (NI) and provides a specialized service for the whole population. Since 2003, the RBHSC pediatric cardiology team has provided a sustainable home monitoring service for children with CHD post discharge from the pediatric cardiology unit [1-11]. This service has provided valuable ongoing contact and follow-up consultations between children and their parents and pediatric cardiology consultants and nurses, and it has reduced the need to travel to the hospital as frequently as would previously have been required. Considerable evaluation of the RBHSC pediatric cardiology remote consultations (RCs) using a videoconference service has already been conducted, and the data demonstrated an effective system providing reliable follow-up for babies or children assessed and monitored using audio and visual technologies [3,10]. This approach has been verified as effective and safe in a systematic review of telemedicine for pediatric cardiology [12]. RCs for pediatric care can offer a more timely response and enhanced quality of care when compared to in-person consultations [13-17]. Parents have reported a reduction in anxiety and an increase in satisfaction with services when receiving RCs compared to hospital consultations [6,10,13,18,19]. The evidence to support the use of telemedicine in general has been increasing over the last 2 decades, and there is now consensus on the benefits to children, their parents, health care staff, and the health service [7,13,20-23].

The COVID-19 pandemic presented major concerns for families regarding bringing children with CHD out of the safety of their home to the hospital, and the risk of exposing them to possible infection during travel or at their hospital appointment. Due to the COVID-19 pandemic, the use of telemedicine has been encouraged [24,25], and RC is ideal for patients, as it reduces the risk of exposure to infection [9]. Therefore, in order to meet the clinical needs of children and protect them and their families, the RBHSC service was expanded to offer remote pediatric cardiology RCs to children aged up to 16 years.

Initially, the RBHSC home monitoring service was facilitated by videoconferencing using Tandberg (Overland Tandberg) and Cisco (Cisco Systems, Inc) devices and software set up in the hospital videoconferencing suite and in the patient’s own home. The device and, if required, broadband were installed in the patient’s home by the Belfast Health and Social Care Trust. However, during the pandemic, engineers could not enter homes due to the risk of infection, and the Tandberg and Cisco infrastructure could no longer be installed in patients’ homes. Therefore, as of April 2020, when in-person consultations were considered high risk, to maintain RCs and provide greater access, the RBHSC and parents of children with CHD started using a secure, videoconferencing platform (Pexip) with the aim of providing optimal RCs to assess, monitor, and manage condition progression during the pandemic.

The videoconferencing platform is accessed via the Pexip software application; can be downloaded to any internet-enabled device, including a smartphone, tablet, or computer; and provides a broad cloud-based, virtual meeting space that can only be accessed using a personal identification number code. The platform has a high level of interoperability, which allows access to users via a broad range of existing platforms. While increased accessibility may result in issues in terms of security, the developers state that the platform uses “formalized internal information security best practices,” complies with ISO/IEC 27001:2103 and ISO /IEC 27701:2019 standards for information security and privacy and is General Data Protection Regulation compliant. It allows efficient bandwidth use regardless of where the meeting participants are located, ensuring high-quality video and audio. However, some people may experience technical challenges with, for example, the software and hardware, resulting in barriers to the adoption and acceptance of RCs [26]. New technologies in the clinical setting must be assessed and monitored to ensure they meet the ongoing needs of children, their parents or carers, and clinical staff. Therefore, the aim of this project was to evaluate parents’ experience, perceived usefulness, usability, and acceptability of using a videoconferencing platform for remote pediatric cardiology consultations during COVID-19.

## Methods

### Study Design

This quasi-experimental cohort study ran from October 6, 2020, until April 27, 2022, and explored qualitative and quantitative outcomes of using a videoconferencing application for the delivery of 8 pediatric cardiology consultations to each patient to augment patient care during the challenges posed by the COVID-19 pandemic. Web-based questionnaires were developed using Qualtrics software. Based on the Unified Theory of Acceptance and Use of Technology (UTAUT) model [27], adapted for testing the videoconferencing application, the usefulness, usability, and acceptability of videoconferencing RCs were investigated. Parents provided feedback on their experiences of using videoconferencing and taking part in the project during interviews facilitated using WhatsApp (Meta Platforms) video calls.

Findings from this study relating to the number of attendances to the hospital, including both initiated and avoided attendances, as a result of RCs using videoconferencing have been presented elsewhere (Gillender et al, in press, December 2024).

### Recruitment

Parents of children diagnosed with CHD were identified in the hospital ward, outpatient departments, or other pediatric cardiology satellite units within NI. Parents were approached by a pediatric cardiology clinician who informed them about the project and provided the participant information packs (PIPs). Parents provided written informed consent via email, WhatsApp, or post to 1 researcher (JEMM).

In order to minimize any risk of cross-contamination with coronavirus, the PIPs were printed and prepared while adhering to a strict protocol whereby a single masked operative, wearing gloves, prepared and packaged the PIPs. PIPs were delivered directly to the hospital where they remained in locked storage until required.

Parent or carers who had a child aged 0‐16 years attending pediatric cardiology at the RBHSC were invited to participate and receive 8 RCs as part of their child’s routine home monitoring care provision. The inclusion criteria included access to a device on which to download the videoconferencing application and ability to understand spoken and written English. The State Trait Anxiety Inventory (STAI) [28] was used as a screening tool, and parents with a score greater than 65 who chose to opt out of the project were excluded.

Parental anxiety levels were measured prior to the first RC and at the end of the project, using a web-based version of the validated self-completion STAI questionnaire [28], which indicates anxiety levels for a single point in time. The average STAI score for working adult females is 35.2 (SD 10.61), and for males, it is 35.72 (SD 10.40); the score is also age dependent [28]. However, given the nature of the medical condition under consideration, anxiety scores above the average for this population were expected. Based on previous research involving parents with children with CHD in NI [29] and a South American validation study of the STAI and the Beck Depression Inventory [30], the mean STAI scores for participants with anxiety was 52.8 (SD 11.42), giving a cutoff score of 65.

### Videoconferencing Application

The videoconferencing platform can be easily accessed via the application, downloaded to any device, and used straight away. In most cases (21/32, 66%), participants were assisted with downloading and testing the application during an in-person discussion with the research doctor (JG). However, if children had already been discharged parents received an instruction sheet via email (Multimedia Appendix 1). RCs were carried out by JG and RB together or separately. Upon completion of the project children who continued to require home monitoring had further RCs.

### Data Collection

Parents completed a baseline questionnaire at enrollment (T0) to collect demographic details and information about their use and experience of technology. Approximately 24 hours before and after the first (T1), fourth (T2), and eighth (T3) RC, parents were asked to complete the UTAUT questionnaire and details about their child’s most recent RC. The UTAUT model is a theoretical framework that seeks to explain and predict an individual’s intention to use a technology, as well as their actual use of that technology [27]. This questionnaire has been validated across a wide range of countries and for assessing different types of technologies [31,32]. Questions related to the quality of the technology interface based on work by Zhang et al [33] were also included. The UTAUT is designed to be adjusted to fit the technology under investigation, and therefore a certain amount of rewording was expected [27].

The UTAUT model is based on several key constructs:

1. Performance expectancy: An individual’s belief that using a technology will lead to improved performance in a specific activity.
2. Effort expectancy: An individual’s belief that using a technology will be easy and require minimal effort.
3. Social influence: The influence of others (eg, family, friends) on an individual’s decision to use a technology.
4. Facilitating conditions: The availability and accessibility of necessary resources (such as training and support) that enable an individual to use a technology.

Table 1 shows the questions used in the project in relation to each construct. As part of the project, if required, parents were supplied with weighing scales and an oxygen saturation monitor to report their child’s weight and oxygen saturation at each RC and for use as required.

**Table 1.** Project-specific questions used in relation to each construct of the Unified Theory of Acceptance and Use of Technology questionnaire.

Construct	Measure item
Performance expectance	Using Pexip speeds up my child’s care Using Pexip makes it easier to manage my child’s care Pexip will increase the quality of my child’s care I find Pexip useful for my child’s care
Effort expectancy	Learning to operate Pexip has been easy for me I find Pexip flexible I find Pexip easy to use Using Pexip takes too much time Using Pexip is complicated Learning to use Pexip has been easy for me Overall, I believe that Pexip is easy to use
Social influence	People who are important to me think that I should use Pexip The hospital has helped me to use Pexip The hospital has supported me to use Pexip
Facilitating conditions	I have control over using Pexip There is someone available to help me with any difficulties I have with Pexip Using Pexip fits well with my and my child’s routine

### Parent Interviews

Parents were invited to discuss their experience of using the videoconferencing application for their children’s routine RCs. Interviews were conducted via WhatsApp by 2 members of the research team (MS and JEMM) using open and closed questions. WhatsApp was used to ensure parents skills and experience relating to the use of the videoconferencing application was strictly confined to the RCs. All interviews were audio recorded and carried out within 1 week following T1, T2, and T3 and at parents’ discretion. Training in the use of video calls was not required, as all parents were experienced users.

Interviews were analyzed using Braun and Clarke’s Reflexive Thematic Analysis method [34]. This approach was considered the most suitable to explore the lived experiences of using videoconferencing and add meaning to the findings from the UTAUT questionnaire.

### Statistical Analysis

Descriptives statistics were generated. Frequencies, means, and SDs were generated according to variable type. Cronbach α’s were generated for constructs (Table 1). Using repeated measures analysis of variance, the pre- and postintervention UTAUT questionnaire data points were analyzed.

### Ethical Considerations

Ethical approval was granted by East of England—Cambridge South Research Ethics Committee on September 23, 2020 (20/EE/0190) and Confirmation and Capability at Belfast Health and Social Care Trust was granted on October 6, 2020 (HSC Trust reference: 20028MS-SW). All parents and children had the choice to opt out of the study or leave the study at any time after providing informed consent, without the need to provide a reason and without otherwise affecting the medical care children received. All quantitative data collected were anonymized, and all qualitative data were deidentified before analysis. In this study, no compensation was provided to parents or children for their participation.

## Results

### Recruitment

**Figure 1.**
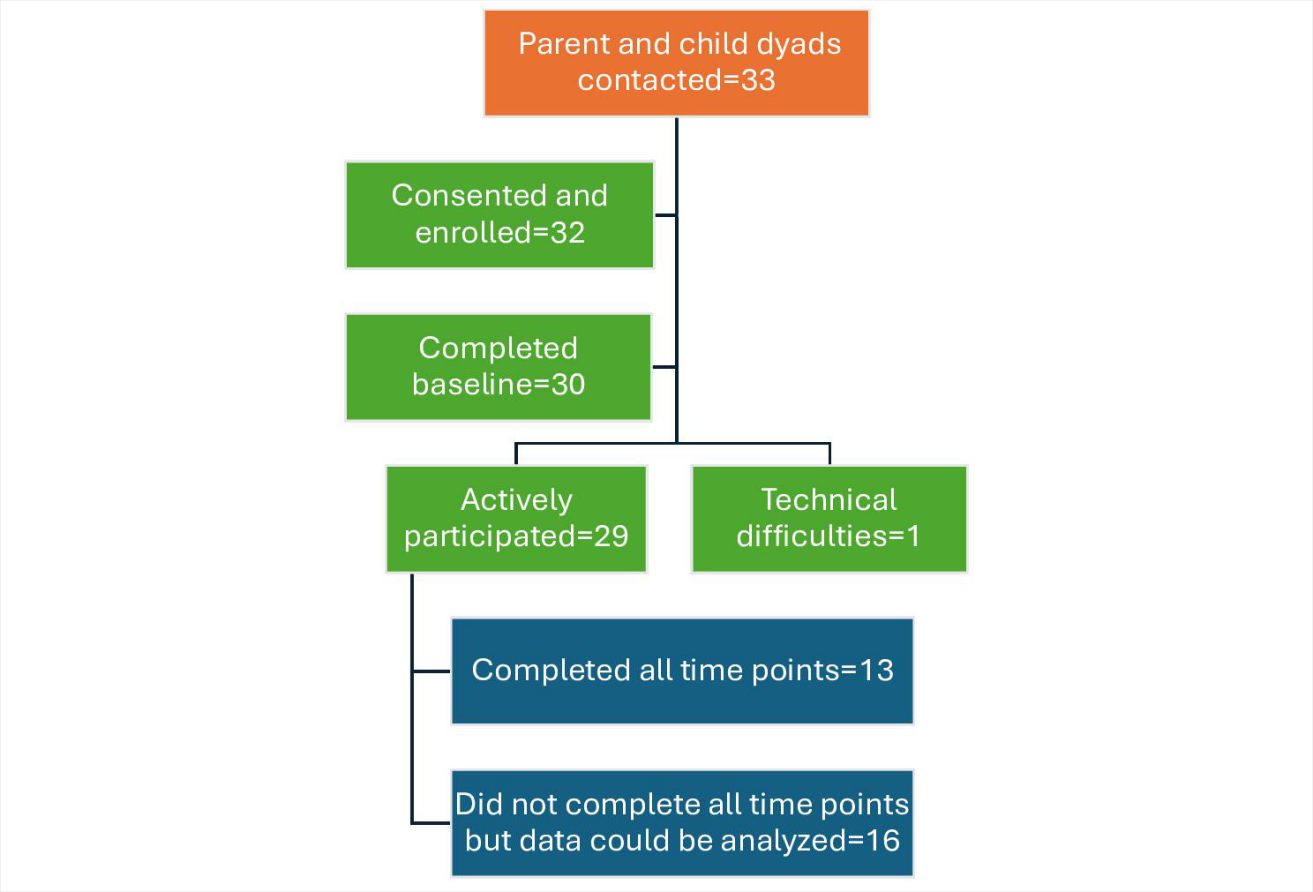
Recruitment and retention flowchart for the pediatric cardiology remote consultation project.

The parents of 33 children were approached while their child was an inpatient at the RBHSC or a regional general hospital (n=21) or was attending an outpatient clinic (n=12), and 32 child and parent dyads provided written consent and were enrolled in the project. For 1 child, both parents actively participated in the project (participants 1004a and 1004b). In total, 30 dyads (31 parents) completed the baseline questionnaire, and 1 dropped out due to technical issues. Therefore, 29 child and parent dyads completed the project, with 13 completing all time points. Figure 1 demonstrates the flow of participants throughout the project.

### Participant Characteristics

Of the parents who completed the project, 93% (n=28) were mothers, 11 of whom were first-time mothers, and 6 (54.5%) of these received their child’s diagnosis post birth. None of the parents had an STAI score above 65. Of the 29 children participating, 38% (n=11) were female. In total, 23 children were aged younger than 3 months at enrollment (mean 37.62, SD 32.5 d), 5 were aged between 3 months and 1 year, and 1 child was over 34 months (1052 d). Families resided across all 5 Health and Social Care Trusts in NI at a maximum distance of 133.1 km from the RBHSC, equating to a 3‐ to 4-hour round trip (Multimedia Appendix 2). Table 2 shows the demographic characteristics of the participating parents and children. The participating children had a range of complex conditions requiring surgery within the first 6 months of life (Table 3). In total, 17 children had a surgery or procedure during their participation. Half of the children had a coexisting congenital anomaly, with Down syndrome being the most common (n=7, 24%).

**Table 2.** Demographics of the parents (n=30) and children (n=29) who completed the pediatric cardiology remote consultation project.

Characteristics		Participants
**Gender (parents), n**		
	Female	28
	Male	2
Age of parents (years), mean (range; SD)		32.9 (20‐47; 5.5)
**Education level of parents, n**		
	No qualifications	0
	High school	6
	College	8
	Apprenticeship	2
	Undergraduate	6
	Postgraduate	8
Distance from home to Royal Belfast Hospital for Sick Children (km), mean (range; SD)		49.3 (2.3‐133.1; 39.61)
**Timing of child’s diagnosis, n**		
	Antenatal	13
	Postnatal	16
**Sex (children** **), n**		
	Female	11
	Male	18
Age of children at enrollment (days), mean (range; SD)		98 (4‐1052; 191.14)

**Table 3.** Conditions of children included in the pediatric cardiology remote consultation project.

Primary cardiac diagnosis	Cases (n=29), n (%)
Tetralogy of Fallot	7 (24)
Atrioventricular septal defect	5 (17)
Ventricular septal defect	4 (14)
Shone complex (days)	2 (7)
Dysplastic tricuspid valve	2 (7)
Coarctation of aorta	2 (7)
Complex single ventricle pathology	2 (7)
Transposition of the great arteries	1 (3)
Patent ductus arteriosus	1 (3)
Total anomalous pulmonary venous drainage	1 (3)
Cardiomyopathy with severe outflow obstruction	1 (3)
Restrictive and hypertrophic cardiomyopathy	1 (3)

### Videoconferencing Enabled Remote Consultations

In total, 201 (range 1‐8) RCs were completed as part of the project, with the first RC (T1) taking place at a mean duration of 25.07 (SD 17.74; range 0‐57) days following discharge.

Data gathered at baseline demonstrated that 23% (7/30) of parents were somewhat or not very experienced in the use of technology (Table 4). However, following their first experience using videoconferencing for RCs (T1) 83% (20/24) agreed that it was easy to use. At T3, 100% (n=17) agreed it was easy to use.

**Table 4.** Participating parents’ (n=30) self-reported level of experience of using technology at baseline (T0).

What is your level of experience in using the following	Not very experienced or somewhat experienced, n (%)	Neither experienced or inexperienced, n (%)	Quite experienced or very experienced, n (%)
Electronic devices such as smart phones and tablets	6 (20)	3 (10)	21 (70)
Computers	9 (30)	3 (10)	18 (60)
Talking to others on video calls	8 (26.7)	3 (10)	19 (63.3)
Technology overall	7 (23.3)	4 (13.3)	19 (63.3)

A total of 63 post-RC questionnaires out of a possible 90 were completed, giving a 70% completion rate. On these occasions, 100% (n=29) of parents agreed or strongly agreed with the question “The problems and or questions I had were answered to my satisfaction during the videoconference.”

During 97% (61/63) of the RCs, parents used smartphones, including iPhone (n=24, 38%) or Android (n=37, 59%) smartphones. For 2 calls, an iPod was used. For 21% (13/63) of the calls, an audio problem was reported; these were resolved on most occasions by hanging up and reconnecting or performing a forced stop of the videoconferencing application and reconnecting. No parents reported an issue with the video quality of the RCs.

### Technology Acceptance

The Cronbach α coefficients for the constructs in the UTAUT adapted for this study were 0.94 for performance expectancy, 0.91 for effort expectancy, 0.76 for social influence, and 0.77 for facilitating conditions, suggesting that the questions presented in the questionnaire were correlated and measured the same concepts.

Across the 4 constructs of the UTAUT, results showed a nonstatistically significant trend upward (n=14). Therefore, this suggests that as parents became more experienced at using videoconferencing for RCs their beliefs in its ease of use and their acceptance of it were enhanced.

### Parents’ Experiences

A total of 41 interviews were conducted via video call with 26/30 parents (Table 5) to assess parents’ experiences. The total duration of interviews analyzed was 19 hours and 56 minutes. Further, 1 parent declined to take part in any interview, and 4 could not take any calls due to their baby being admitted to hospital, work commitments, and scheduling issues. In total, 5 participants enrolled in the project completed all 3 interviews. Parents did the interviews most often with their child; alone; or with other family members present, including their child’s other parent, sibling, or grandparent. On average, interviews lasted 20 minutes. Data saturation was achieved.

**Table 5.** Number of parents (n=30) completing video call interviews on the pediatric cardiology remote consultation project.

Video call interviews	Parents completing interviews (n)
No interview	5
1 interview	14
2 interviews	6
3 interviews	5

Parents reported their main concerns discussed at each RC were feeding, including breast feeding; weight; medications; surgery timing; surgical wounds; and details about their child’s condition. Further, 1 parent reported that they asked about childhood vaccinations and about taking their baby swimming. At T1 (n=23) and T2 (n=20), 100% of parents agreed their problems or questions were answered to their satisfaction during the RC, and 94% (15/16) of parents agreed at T3. Following the RCs, parents agreed it had been beneficial at T1 (19/23, 83%), T2 (20/21, 95%), and T3 (13/17, 76.5%).

An experienced member of the pediatric cardiology team assessed key clinical parameters during each RC. These included the child’s feeding, weight, medications, color, breathing, oxygen saturation, and overall well-being. The clinical outcomes from this study are reported separately (Gillender et al, in press, December 2024).

Of the 26 parents who were interviewed 100% felt using videoconferencing for RCs was helpful, were pleased to have the opportunity to use it, and would recommend it to others. Benefits included reduced time and cost for the family in terms of travel, fuel, and childcare costs, having a planned call with a pediatric cardiology specialist at a time that worked for them and their family, and the flexibility of the RC.

Thematic analysis of the post-RC video call interviews using Braun and Clarke’s approach [34] identified 5 key themes:

- The ease of using videoconferencing for RCs
- The reassurance of having regular RCs
- The value of the visual component of the RCs
- The opportunity to ask questions during RCs
- The non–health-related benefits of RCs

## Discussion

### Principal Findings

Parents caring for sick children are vulnerable to “social, psychological, physical, relational, individual, and financial” risks [35], which can lead to detrimental effects on the well-being of their children and other family members [36]. However, caring for children at home can have positive effects for the child, parents, and wider family, and strong, trusting relationships between parents and the clinical care team may reduce parental stress levels and contribute to resilience [37,38].

Remote patient monitoring has become more prevalent and relevant as the pressures on health care providers and service delivery increase. However, RC can only provide sustainable and optimal health care when considered within the reality of the patient lived experience; clinical expertise; and available finances, services, and resources.

In this project, over half of the parents had a baby whose CHD diagnosis was made postnatally. Navigating the complexities of new parenthood, an unexpected diagnosis, and new methods of interacting with health professionals using technology, all during the uncertainty of the COVID-19 pandemic, led to parents feeling frightened with deep concerns about all aspects of their child’s health.

*I was frightened, it’s the only reason I said I would go home, if I had something like that [videoconferencing for RC], if they give me contact with somebody*

[Mother 1027]

### Parents’ Experiences of Videoconferencing Enabled Remote Consultations to Manage Their Child’s CHD

#### The Ease of Using Videoconferencing for Remote Consultations

Findings from the UTAUT questionnaire showed that using videoconferencing for RCs got easier over time and became more acceptable. Parents found the informal coaching in downloading and using the application to be very beneficial and the written instructions easy to follow. Although 27% (8/30) of parents reported limited experience of talking to others on video calls, they reported that they found the videoconferencing application easy to use, suggesting they would be more likely to connect.

*You literally just click on the app, it’s…really straightforward, I called him [doctor] and it worked…it’s very simple*

[Mother 1010]

Parents quickly became experienced using videoconferencing for RCs and meeting with the clinical team. They were better prepared for the calls, actively made decisions to improve the interactions, and set up in a way that worked best for them. For example, having their baby undressed for visual assessment, weighing their baby in advance, and having the oxygen saturation monitor nearby or attached to the baby. In addition, if there was an issue with the audio or video quality during a RC they reconnected to resolve the issues or used a forced stop of the videoconferencing application. As their skill and knowledge increased, their confidence increased, and they found it easier to ask questions.

*Before I went on the calls I was a wee bit anxious…I didn't know what I was expecting, but it actually made me feel quite at ease because I know now that…every two weeks we do it*

[Mother 1023]

#### The Reassurance of Having Regular Remote Consultations

Becoming a parent can have major physical, social, and psychological implications [39], and new parents require help and support from family and friends to successfully navigate their new role and responsibilities [40]. However, when their baby has a complex medical condition, support and guidance are also required from specialist medical teams, as well as building strong relationships to optimize the child’s care using the most appropriate techniques and technologies [35,41]. Remote monitoring, while essentially about the patient, also supports parents’ emotional well-being and empowers them which is essential for the continued and sustained welfare of their children.

*I think it’s great to get the confidence to be able to take the lead in the child’s care and obviously have that support in the background. I think sometime parents can be overwhelmed by medical staff and they might think they’re not doing things right, but a parent’s intuition is always the best intuition I think*

[Mother 1004]

#### The Value of the Visual Component of the Remote Consultations

This project aimed to provide the highest quality of interactions via synchronous RCs. Parents were impressed by the quality of the video and felt that the visual aspect was key for them. Theoretically, “ocularcentrism,” where people need to see each other to enable them to communicate more effectively, is likely an important determinant in their preference to use videoconferencing for RC [42].

*Describing symptoms over the phone isn't that useful because they ask you what colour he is and it’s hard to describe a colour over the phone, it’s hard to describe his breathing over the phone, it’s so much better if they can just actually see him and look at him…the seeing bit is key*

[Mother 1001]

RCs allow parents to care for their child at home while ensuring very sick children are rapidly admitted to a hospital when required [16]. The RBHSC team was experienced in carrying out RCs and in using the video and audio to assess babies and children, and parents trusted the doctor and the continuity of care.

*I thought she [baby] just had reflux but it was [doctor] on the call made the call, calm as you like, “I'll see you in the Royal [RBHSC] in an hour”. And I was like, why is he dramatizing this?… Of course then when we arrived…that was that, she was going for a stent. But I didn't realise she was sick, because you know, to me she’s not blue, although she is blue to other people…. So I think, because I'm constantly looking at her, I didn't realise she was sick and I was blaming the machine [oxygen saturation monitor] and he was like “I think maybe you should just come on up,” and I was thinking why’s he taking me up that road?… and she was admitted…so without this [RC] I would never have ever in a million years ever phoned and said she’s off form because for me it was reflux and I just thought she'll be fine…. When I got up there, I realised the extent of it, I totally owe her life to him…he was so calm, I just thought he was being dramatic, you know really checking her out…he saved her that day, he definitely did*

[Mother 1003]

#### Opportunity to Ask Questions During Remote Consultations

In a web-based survey, 90% (n=10) of parents of children with CHD reported that the question *How many children have heart surgery and how many survive?* was important or really important to them [29]. Parents have many questions regarding their child’s condition but often find it difficult to have the confidence or be “brave” enough to ask.

*it gives me massive confidence, massive…as I say I wouldn’t be panicky anyway but I’ve never had a cardiac baby before…. I do tend to have a lot of questions*

[Mother 1024]

#### Non–Health-Related Benefits of Remote Consultations

Many parents simply cannot stay at the hospital with their sick child for long periods of time. They feel they neglect their other children, do not have time to travel long distances to the hospital, or do not have the finances or support infrastructure to do so, exacerbating their feelings of stress and anxiety. RC is convenient, as it reduces costs, time, childcare, travel, preparation, and waiting for children and their parents, especially for those in remote areas [15,19,43-46]. Parents in this project agreed with this.

*it’s just so convenient especially with home schooling at the minute because the three of them [other children] are at home…it saves me round trips so it therefore saves you on your pocket, it saves you time because it’s not just an hour up the road and down the road, you have to consider your parking…then your appointment takes maybe three hours it’s a whole day, a whole day, its mad!*

[Mother 1003]

Flexibility of the timing of the RCs was important to parents to enable them to have some time to prepare for the call, and the overall convenience improved parents’, and therefore their child’s, experience. It also facilitated streamlined RCs and improved use of time for the clinicians.

*It’s so flexible which is brilliant it’s not like having to be at the hospital for a certain time with parking and everything that goes with it…it would be half a day if we had to go to the hospital because getting packed up and all his stuff and medications, getting to the hospital, getting parked, getting in, and the phone call was 15, 20 minutes*

[Mother 1019]

#### COVID-19

In response to the COVID-19 pandemic, the General Medical Council (GMC) [47] recommended that “doctors should...triage and treat patients by remote consultations where possible,” and the Royal College of Physicians [48] stated “there has never been a time when effective remote consultations have been needed more.” The number of visits and in-person consultations at the RBHSC were reduced during this project, therefore reducing the risk of infection for vulnerable children. Interestingly, however, COVID was only mentioned on 4 occasions by 4 parents. Further, 3 were not that concerned about COVID.

Additionally, 1 parent was concerned that their child could “catch something” while at the hospital. Nevertheless, they still wanted their child to have in-person consultations at the hospital.

*if you’re going up there [RBHSC] every week then obviously there’s a chance…that something could happen or she could catch something…but I still like going up every so often just for that wee bit extra reassurance*

[Mother 1013]

### Comparison to Tandberg

The necessary move away from the Tandberg and Cisco remote monitoring system to the Pexip application RCs meant no installation of hardware was required. The application could be downloaded to any device, and RCs could begin immediately once children left the hospital allowing them to be discharged sooner. Compared to static Tandberg, the videoconferencing application was superior in terms of the cost of hardware installation and convenience, bed availability, carbon footprint, and potentially workforce sustainability. By using mobile phones, parents could zoom in on their baby, enabling the clinicians to visually assess the baby. The health care providers could also see the surroundings of the babies, which may be an important tool if there were concerns about the home environment. In 2018, the GMC [49] published a report commissioned to inform the regulatory requirements relating to telemedicine in the United Kingdom, with the key focus being to ensure that patients’ safety was not compromised. The GMC [47] provided a flowchart to assist clinicians to “weigh up the factors” regarding whether to provide treatment remotely or in person. However, this guidance does not mention the technological tools and skills required of the patient or carer. The reported usefulness, usability, and acceptance of the videoconferencing application for RCs by parents suggest it would be rapidly adopted by parents who quickly and easily learn to use the application, set up to optimize the call, and troubleshoot, making it as easy as possible for them to connect and engage with clinicians.

Managing and monitoring the care of sick children are the main focus for clinical teams during RCs. However, they also have the potential to provide reassurance and support to parents by reducing their anxiety and giving them a vital role in the care and decisions regarding their own baby at a time when many may be overwhelmed by a CHD diagnosis. In a project in China, Zhang et al [50] used the WeChat platform for remote monitoring of infants with CHD. It included a clinician led live daily interactive question and answer session for parents and led to a reduction in parents’ depression and anxiety [50].

### Limitations

It is important to remember that while NI has the highest rates on fiber broadband availability in the United Kingdom, a proportion of the population have poor internet access, mainly homes in rural areas [51] both in NI and worldwide. However, no child should be disadvantaged by a lack of internet, and continued support, as required, from the RBHSC and in other jurisdictions in terms of hardware and software is warranted. Such provision should be viewed as an investment, not a cost. Worldwide, key to the success of RC provision is parent and carer involvement, including training, guidance, and support to enhance digital literacy and confidence in using technology.

Throughout the project, parents and the RBHSC pediatric cardiology team were clear that RCs should not and would not replace in-person consultations. Although they reported RCs were as good, parents still valued the hands-on, in-person consultations above RCs. Therefore, and as shown in this current project, a hybrid model of RCs and in-person consultations based on patient needs would provide the best possible care. However, the duration between in-person appointments may be lengthened if parents find this acceptable and children are well.

Caution is needed when using RCs for remote monitoring of children. Not all parents were regularly available for RCs, and it was suggested by 1 parent that some could use remote monitoring in negative ways by not disclosing details about the child’s health if they faced financial or emotional difficulties.

*I*n-person *I know they’ll not let me go home if she’s not right whereas, if you were a wee bit careless you could mask what’s going on…because you can't be bothered doing the [trip to the hospital] or the other thing is, probably if you struggled financially…maybe it’s just not possible to do the trip so maybe you're going to tell lies*

[Mother 1003]

As clinicians continue to use RCs more for remote monitoring, they are at risk of experiencing digital fatigue, ”a state of mental exhaustion and disengagement caused due to prolonged exposure to digital tools, apps, and screens” [52]. Therefore, it is important to protect clinicians who are already under unprecedented levels of pressure as patient numbers continue to increase. It is also important that for clinicians who are new to RCs or are using a new software for RC, extra support and time are provided to develop their skills in identifying issues and using the technology.

### Future Opportunities

As remote monitoring becomes more prevalent, future opportunities for additional functionality may include artificial intelligence (AI) and machine learning to enhance engagement and reduce workload. AI could be incorporated to schedule appointments, and natural language processing could be used to automatically parse and interpret RC dialog to provide personalized reminders and follow-up tasks. Machine learning algorithms could be embedded for continuous monitoring of and real-time feedback on data from oxygen saturation monitors, to identify high-risk patients and target interventions.

## Supplementary material

10.2196/54598 Multimedia Appendix 1 Pexip application download instruction sheet provided to participating parents.

10.2196/54598 Multimedia Appendix 2 Map of Northern Ireland showing the home locations (blue) of participating child and parent dyads in relation to the Royal Belfast Hospital for Sick Children (red).

## Abbreviations

AI: artificial intelligence
CHD: congenital heart disease
GMC: General Medical Council
NI: Northern Ireland
PIP: participant information pack
RBHSC: Royal Belfast Hospital for Sick Children
RC: remote consultation
STAI: State Trait Anxiety Inventory
UTAUT: Unified Theory of Acceptance and Use of Technology
