# Supplementary material for: Touching Technology—Parents’ Experiences of Remote Consultations for Children With Severe Congenital Cardiac Conditions: Quasi-Experimental Cohort Study
Source: JMIR Pediatr Parent. 2024 Oct 22;7:e54598. doi: 10.2196/54598 (PMC11521195; doi:10.2196/54598)

- Download ‘PEXIP Infinity Connect’ App on App store or Google Play store


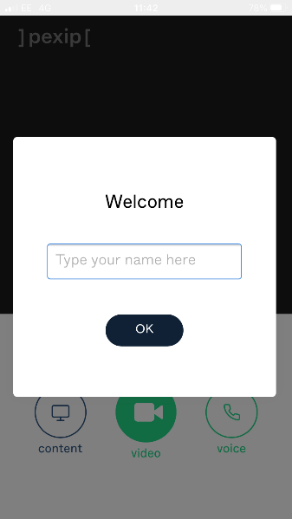

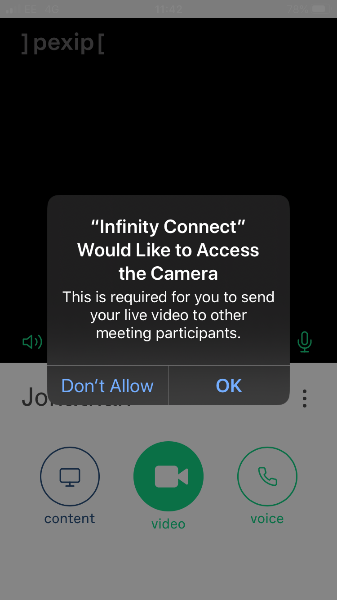

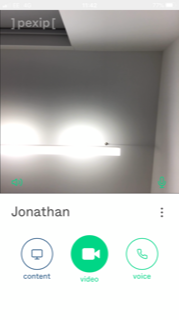

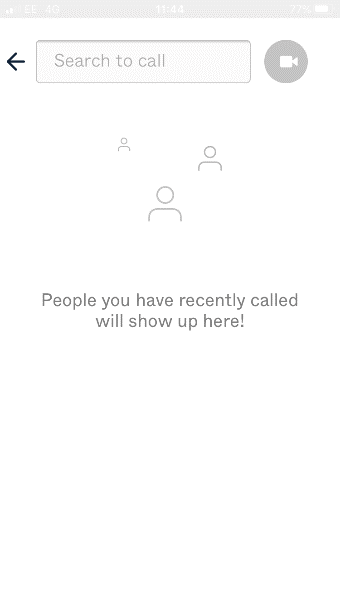


Press the video button to make the call

Press OK to use the camera function

Type in your name and press OK

In this search bar type

6110032@hscni.net


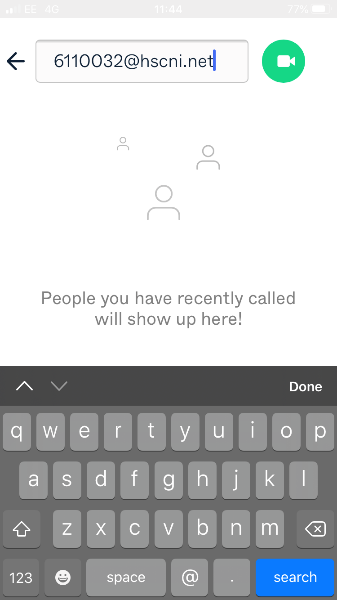

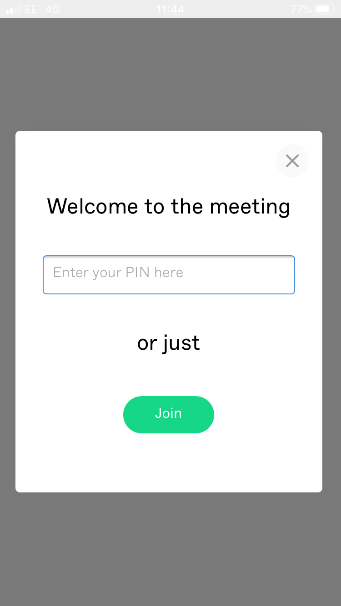

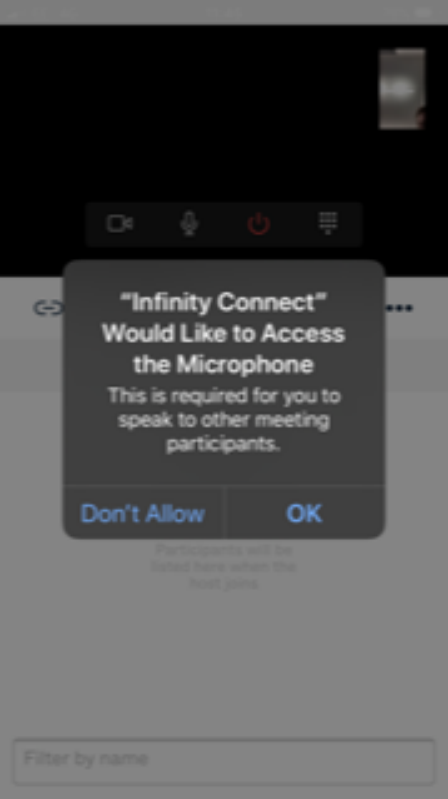


Press Ok to allow the App to access the microphone

Press Join to join the meeting. You do not need to enter a PIN

Press the video button once the email address has been entered


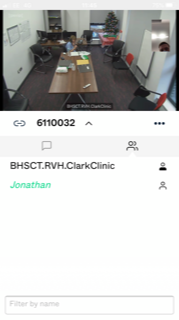


You will now be connected to the videoconference. Your name will appear below the Clark clinic address in green.

To change the settings of the camera (front/rear facing camera, press the 3 dotted icon in the top right hand side circled in red.

This will bring you to control settings. Press ‘Select media devices’ to access further options.

The menu will have 3 options

1 Default camera 🡪 allows you to choose between front and rear camera on your device

2 Default microphone (no need to change)

3 Default speaker (no need to change)


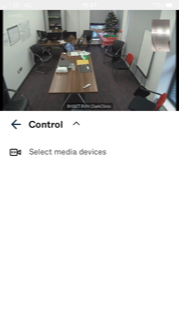

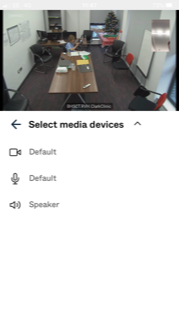

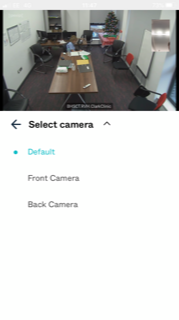

Supplement: Multimedia Appendix 1 [file pediatrics-v7-e54598-s001.docx]
