# Supplementary figures and images for: Touching Technology—Parents’ Experiences of Remote Consultations for Children With Severe Congenital Cardiac Conditions: Quasi-Experimental Cohort Study
Source: JMIR Pediatr Parent. 2024 Oct 22;7:e54598. doi: 10.2196/54598 (PMC11521195; doi:10.2196/54598)

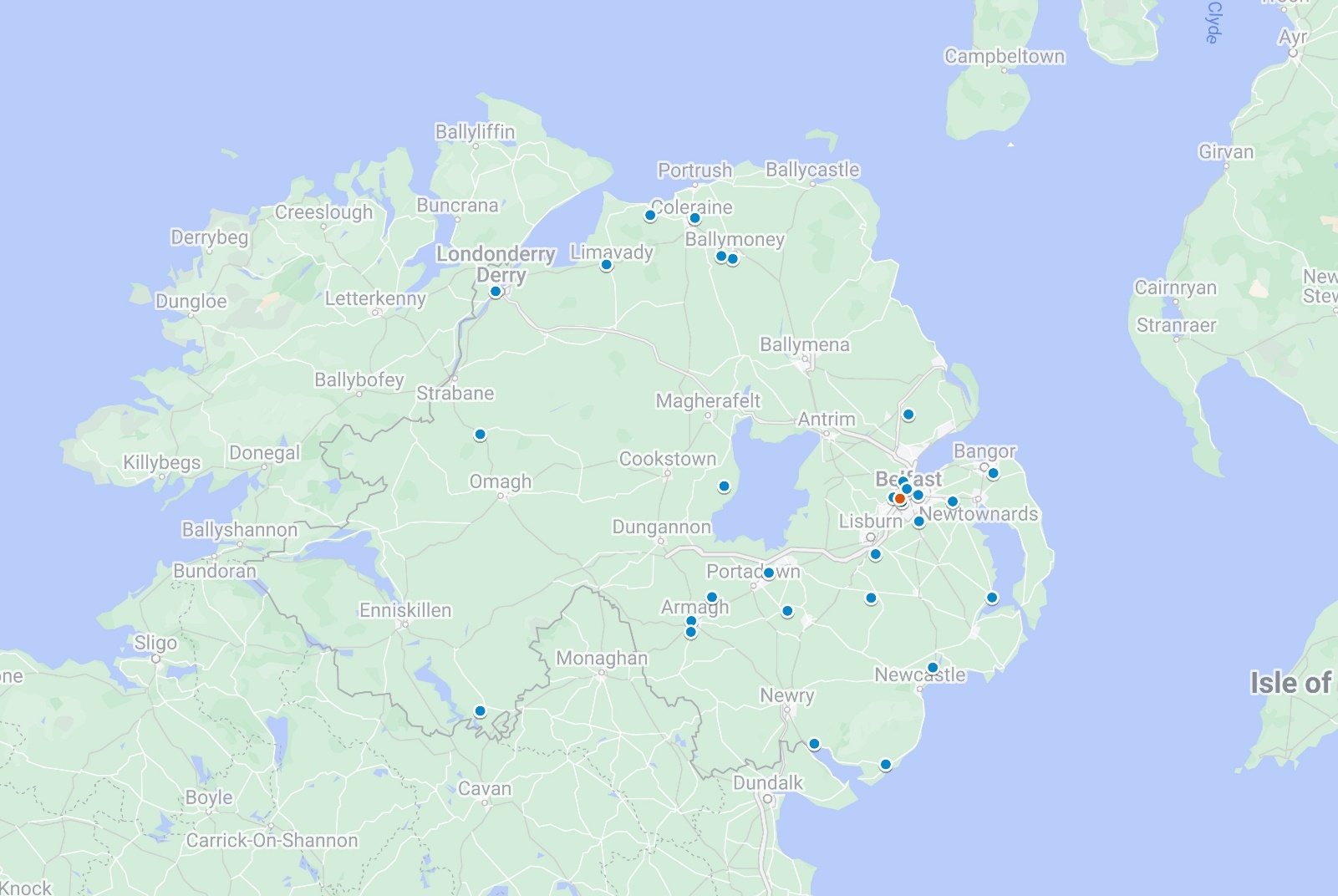

Supplement: Multimedia Appendix 2 [file pediatrics-v7-e54598-s002.docx]
